# Assessment of the pre-emptive effect of photobiomodulation in the postoperative period of impacted lower third molar extractions: A randomized, controlled, double-blind study protocol

**DOI:** 10.1371/journal.pone.0300136

**Published:** 2024-06-17

**Authors:** Daniel Rodríguez Salaberry, Laura Hermida Bruno, Rolf Wilhem Consolandich Cirisola, Priscila Larcher Longo, Maria Cristina Chavantes, Ricardo Scarparo Navarro, Marcela Letícia Leal Gonçalves, Ana Paula Taboada Sobral, Thais Gimenez, Cinthya Cosme Gutierrez Duran, Lara Jansiski Motta, Sandra Kalil Bussadori, Anna Carolina Ratto Tempestini Horliana, Raquel Agnelli Mesquita Ferrari, Kristianne Porta Santos Fernandes

**Affiliations:** 1 Postgraduate Program in Biophotonics Medicine, Nove de Julho University, Sao Paulo, Sao Paulo, Brazil; 2 Dentistry School, Universidad Católica del Uruguay, Montevideo, Montevideo, Uruguay; 3 Postgraduate Program in Aging Science, São Judas Tadeu University, Sao Paulo, Sao Paulo, Brazil; 4 Postgraduate Program in Bioengineering, Brasil University, Sao Paulo, Sao Paulo, Brazil; 5 Postgraduate Program in Health and Environment, Metropolitana de Santos University, Santos, Sao Paulo, Brazil; 6 Postgraduate Program in Rehabilitation Sciences, Nove de Julho University, Sao Paulo, Sao Paulo, Brazil; Massachusetts General Hospital, UNITED STATES

## Abstract

Photobiomodulation is a safe option for controlling pain, edema, and trismus when applied postoperatively in third molar surgery. However, administration prior to surgery has been under-explored. This study aims to explore the effectiveness of pre-emptive photobiomodulation in reducing postoperative edema in impacted lower third molar extractions. Two groups of healthy individuals undergoing tooth extraction will be randomly assigned: Control group receiving pre-emptive corticosteroid and simulated photobiomodulation, and Photobiomodulation Group receiving intraoral low-intensity laser and extraoral LED cluster application. The primary outcome will be postoperative edema after 48 h. The secondary outcomes will be pain, trismus dysphagia, and analgesic intake (paracetamol). These outcomes will be assessed at baseline as well as two and seven days after surgery. Adverse effects will be recorded. Data will be presented as means ± SD and a p-value < 0.05 will be indicative of statistical significance.

## Introduction

The extraction of impacted lower third molars is a very common procedure that has been technically standardized since the early 20^th^ century [1]. However, it remains associated with a high degree of postoperative discomfort, primarily related to pain, swelling, trismus, and dysphagia [1, 2].

To minimize these unpleasant effects on patients’ quality of life, various pharmacological and non-pharmacological measures with varying degrees of clinical effectiveness have been recommended [1, 3]. Pharmacological measures include the use of analgesics, antibiotics, and anti-inflammatory drugs, primarily corticosteroids [1, 3–7]. Some authors have suggested the pre-emptive use of anti-inflammatory drugs for controlling postoperative pain and swelling following third molar extractions [8–11]. However, there is no definitive evidence with regards to the benefits of prophylactic use, which has been associated with numerous side effects, such as hyperglycemia, insulin resistance, the development of glucocorticoid resistance, hypertension, muscle atrophy, lipid deposition abnormalities, interference with normal healing, an increased risk of infection, mood disorders, cardiovascular complications, and osteoporosis [12–14]. Therefore, the restriction of the use of such drugs is essential, especially in prolonged periods [14, 15]. Non-pharmacological resources include cryotherapy, ozone therapy, growth factors, and photobiomodulation [1, 16].

Photobiomodulation has proved to be a viable option when applied postoperatively in third molar surgeries to control pain, swelling, and trismus, both with wavelengths in the red and infrared spectrum when used separately [17, 18]. Thus far, however, the combined use of wavelengths in both ranges of the light spectrum prior to surgery has not been sufficiently investigated [19, 20]. Given the promising results demonstrated by photobiomodulation, probably the preoperative application of red and infrared laser and LED (both extraoral and intraoral) is expected to be effective in reducing postoperative swelling in retained lower third molar extractions.

Thus, the aim of the proposed study is to investigate the effectiveness of pre-emptive photobiomodulation in reducing post-operative edema following the extraction of impacted lower third molars.

## Methods

### Study design

This randomized, controlled, double-blind clinical protocol is in accordance with the criteria for clinical study design as per the SPIRIT Statement. It has been submitted to the Human Research Ethics Committee of the Catholic University of Uruguay (process: 220420). After a verbal and written explanation of the study, volunteers who agree to participate will sign a statement of informed consent. All patients will have the option to withdraw from the study at any time. The study will be conducted in accordance with the Declaration of Helsinki. The treatments will be carried out at the Dental Surgical Clinic of the University Health Clinic (Apolônia) at the Catholic University of Uruguay from November 2023, to July 2024. Any complications or changes will be investigated and reported to the Research Ethics Committee. The project has been registered on the Clinical Trials platform (www.clinicaltrial.gov), registration number: NCT05924191. The data resulting from its development will be stored in an anonymized manner at the Open Science Framework repository. The files will be posted on the already created link (https://doi.org/10.17605/OSF.IO/3EVMR).

### Sample size calculation

The sample size was calculated to provide an 80% power (α = 0.05). The sample size will consist of 30 surgeries per group. Thirty patients with bilateral and symmetric lower third molars (right and left) will be selected. The procedure will then be performed randomly (split-mouth design), totaling 60 surgeries (30 surgeries in the control group and 30 surgeries in the experimental group). For the calculation, we used the 48-hour time point in the article by Aras and Güngörmüş (2010) [21]. The average edema measurement was 105 mm in the control group and 109 mm in the experimental group. The larger standard deviation between the two means ±5 mm was chosen for the sample size calculation.

### Calibration and examiner training

The examiner who will conduct the pre-operative measurements (considered the gold standard) will lead the calibration process for the researchers who will perform the post-operative assessments. Joint training and calibration exercises will be carried out three times, and the data will be discussed among these researchers and the gold standard examiner to achieve an acceptable level of agreement using the Intraclass Correlation Coefficient (ICC) test [22]. Subsequently, the examiners will individually perform the proposed assessments in the study on 10 adult volunteers at different times. These patients will not be part of the sample. Reliability will be determined using the intraclass correlation coefficient (ICC) [22].

### Laser’s safety methodology

A course conducted by qualified professionals will provide comprehensive laser safety training for all researchers, ensuring the safe use of lasers. The laser devices in this study are Class 3b (power varies between 5–500 mW). This category of lasers can cause accidental harm if looked at directly. All participants and researchers will use protective eyewear during the applications and simulations.

### Participant characterization

Participants referred to the Oral and Maxillofacial Surgery and Traumatology service of the Catholic University of Uruguay, who need to undergo the extraction of impacted lower third molars, will be assessed. The selection of cases will be based on the surgical difficulty of the procedure and anatomical position. The molars will be classified according to Pell and Gregory Classes II or III and/or B or C; vertical or mesioangular position, following Winter’s classification, or Class II with the need for osteotomy, or Class III with the need for osteotomy and tooth sectioning, based on the Prant Scale modified by Amarillas-Escobar et al (2010) [23], which is used to assess the difficulty of the dental extraction procedures.

Individuals eligible for inclusion*—*will need to have two impacted lower third molars, with inclusion based on the surgical difficulty of the procedure and anatomical position as described in the Participant Characterization section. There should be a clear indication for third molar extraction, whether due to recurrent infections, malposition, orthodontic recommendation, or a professional recommendation provided in writing. Male and female individuals between 18 and 50 years of age without any comorbidities will be eligible. The individuals should also maintain good oral hygiene to be considered for the study.

Exclusion of individuals—if they have local conditions that contraindicate surgical intervention or could complicate the post-operative period, such as acute pericoronitis in the previous 30 days or temporomandibular joint ankylosis. Smokers, individuals lacking both upper and lower central incisors, those with a history of photosensitivity, pregnant or lactating women, and individuals using anti-inflammatory drugs or analgesics and those with allergies to any drugs used in the study (e.g., paracetamol, 2% chlorhexidine, local anesthetics, sodium bisulfite) will also be excluded. Individuals experiencing surgical complications, such as bleeding or operative difficulties, will be ineligible for the study due to the potential impact on maintaining the expected surgical standards and the accuracy of the study outcomes. Participants undergoing surgical procedures lasting longer than 90 minutes will be excluded from the study. Surgeries lasting longer than 90 minutes could potentially impact the accuracy and reliability of the findings due to the likelihood of increased post-operative trauma, which may result in more significant edema and pain.

### Randomization

We will use a random sequence generator program (https://www.sealedenvelope.com/) to assign participants to the two experimental groups. Randomization will involve 30 participants. Each participant will undergo two surgeries–one on each side. The first surgery will be performed on the right side and treatment for the left side will be the opposite of the randomized treatment, ensuring no repetition of identical treatments in the same patient. A third party not otherwise involved in the study will handle sequence generation and envelope preparation. Opaque envelopes labeled with sequential numbers will contain a sheet specifying the group for the first surgery based on the generated sequence. These envelopes will remain sealed and stored securely in numerical order until the surgeries.

The patients will undergo assessments by the surgeons and, upon meeting the previously described eligibility criteria, will be enrolled in the study. All participants will be submitted to the same surgical protocol. Just prior to the surgeries, the researcher responsible for photobiomodulation application will open an envelope (without changing the sequence of the remaining envelopes) and perform the procedure indicated.

### Blinding of the study

Only the researcher responsible for administering the treatments (who will open the randomization envelopes) will know which treatment is assigned to each side of the patient. Group identities will be revealed after the statistical analysis of the data to all those involved in the study. Therefore, the researcher responsible for data collection, patient, and statistician will be blinded to the treatments assigned to the groups.

Surgery will be performed conventionally, with the LED/laser turned off in the control group. However, the same points will be treated in the pre-operative (baseline) period as in the experimental group. To prevent the patient from identifying non-activation based on sound of the device, the sound will be recorded and played during the simulation. This blinding method ensures that the participants, data collectors, and statistician remain unaware of the treatment assignments throughout the study. The main investigator will obtain informed consent from potential trial participants. Procedures for collecting, sharing, and safeguarding personal information of potential and enrolled participants will be implemented to ensure confidentiality throughout the trial, both during and after its completion.

### Preoperative assessment

All participants who meet the eligibility criteria for the study and sign the statement of informed consent will undergo a preoperative assessment, which will involve the initial measurement (baseline, pre-surgery) of all study outcomes. These assessments will be conducted by a trained researcher for objective outcomes and a calibrated researcher for subjective outcomes. Surgery will proceed as usual, similar to conventional third molar surgery.

### Surgical procedures

One hour after photobiomodulation (t = 0), the participants will undergo the removal of the third molar by a surgeon following the standard technique at the Catholic University of Uruguay.

A local anesthetic will be administered to the inferior alveolar, lingual, and buccal nerves using two 1.8 ml carpules containing 2% mepivacaine with epinephrine 1:100,000 (MEPIADRE 100, DFL, Brazil). An intrasulcular incision will be made with a No. 15 scalpel blade (BIOSET, China) on the buccal side of the second lower molar, extending distally and laterally toward the anterior edge of the mandibular ascending ramus. A triangular periosteal mucosal flap will be dissected using a Molt No. 9 periosteal elevator (HU-FRIEDY, USA).

The buccal and distal bone will be removed using a No. 8 round tungsten carbide bur (STRAUSS AND CO., Israel) and low-speed surgical micromotor (BIOMET 3i, OSSEOCISION NE 111, Japan), with abundant irrigation using 0.95% saline solution. If necessary, coronal and/or interradicular tooth sectioning will be performed using a No. 703 tungsten carbide bur (STRAUSS AND CO., Israel) at low speed and with constant irrigation.

After extraction, thorough cleaning of the residual surgical cavity will be performed, followed by repositioning, and suturing the mucosal flap with 3–0 silk thread (BESTCARE, Portugal) mounted on a disposable circular-section needle (BESTCARE, Portugal).

### Medication protocols in the preoperative period

Control Group: Dexamethasone 8 mg, 1 tablet administered 1 hour before surgery.

Experimental Group: Photobiomodulation applied intraorally and extraorally 1 hour before surgery (detailed in the "Photobiomodulation method" section).

Medication protocols in the postoperative period (both groups):

Amoxicillin 750 mg tablet, orally every 12 hours for 7 days.

Anti-inflammatory medication: Ketorolac 10 mg every 6 hours for 3 days.

Analgesic medication: Paracetamol 500 mg every 6 hours for 3 days to be taken in the occurrence of pain (number of tablets taken will be quantified as a secondary outcome).

### Experimental design

**Fig 1:**
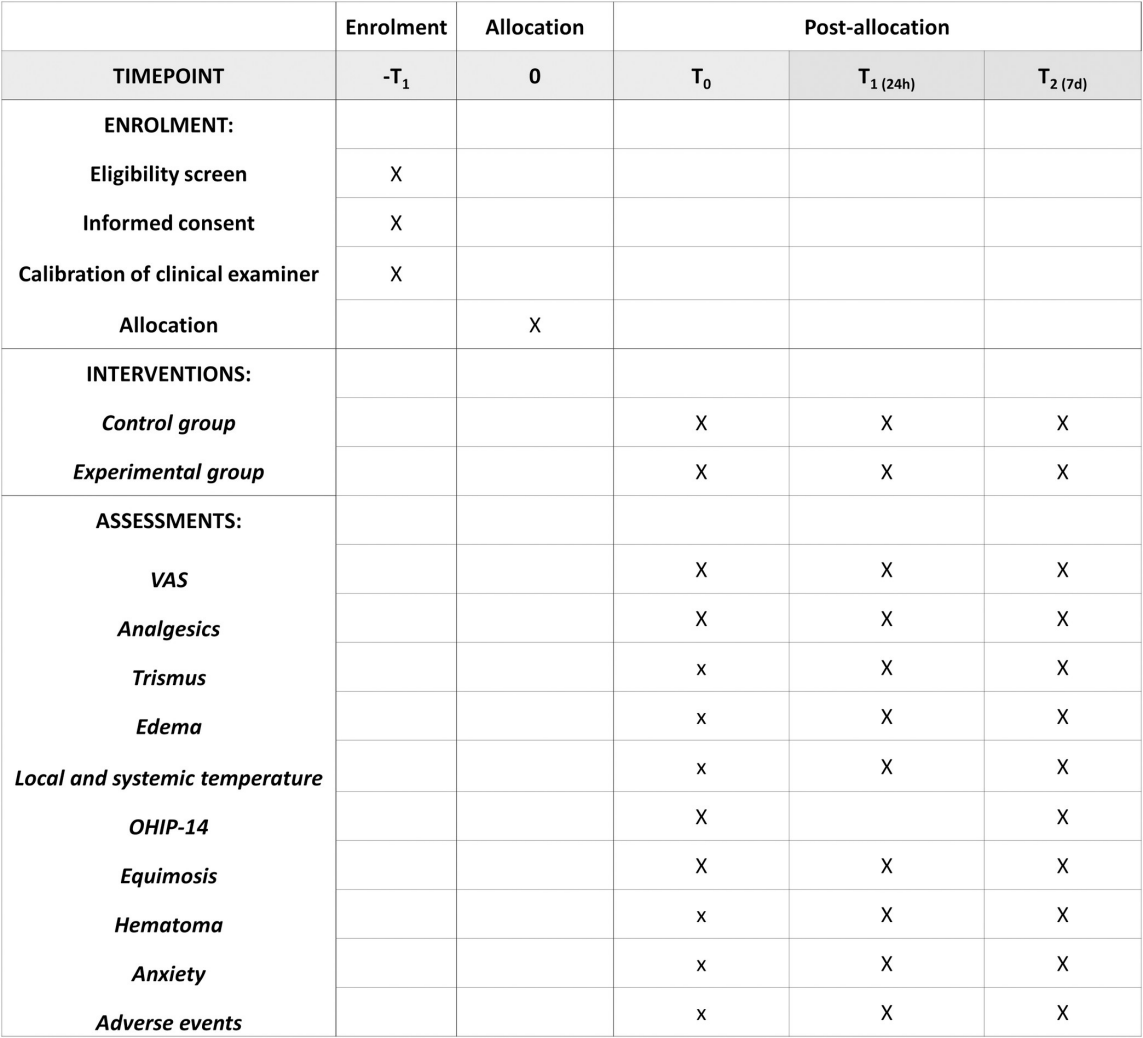
Flowchart of study. VAS—visual analog scale.

Prior to the surgical procedure, the researcher responsible for treatment will open an envelope (without altering the numerical sequence of the remaining envelopes) and perform the procedure indicated. The 30 individuals will be allocated to the experimental and control groups as follows (Fig 1):

Control Group (n = 30 surgeries)—Individuals will receive conventional treatment with dexamethasone 8 mg orally 1 hour prior to surgery [13] + simulated photobiomodulation. The laser will be turned off and will be applied at the same points as in the experimental group in the immediate preoperative period (baseline).

Experimental Group (n = 30 surgeries)*—*Participants will receive photobiomodulation 1 hour prior to the surgical procedure + a placebo tablet of dexamethasone (Laboratory Matías Gonzalez, Montevideo).

Photobiomodulation method—All participants and researchers will wear individual protective glasses during the applications and simulations.

Intraoral photobiomodulation—In the photobiomodulation Group, intraoral irradiations will be performed with a low-intensity laser at a wavelength of 660nm (power of 0.1W, radiant exposure of 1061 J/cm^2^, and energy of 3 J per point, with a 30-second duration at each point, totaling 12J). The same four points will also be irradiated using a wavelength of 808nm (power of 0.1W, radiant exposure of 1061 J/cm^2^, and energy of 3 J per point, with a 30-second duration at each point, totaling 12J), adapted from Sierra et al., 2015 [24].

Photobiomodulation method

All participants and researchers will use protective eyewear during the applications and simulations.

Intraoral photobiomodulation—In the photobiomodulation group, intraoral irradiation (4 points) will be performed with a low-intensity laser at a wavelength of 660 nm (power of 0.1 W, radiant exposure of 1061 J/cm^2^, and energy of 3 J per point, with a 30-second duration at each point, totaling 12 J). The same four points will also be irradiated using a wavelength of 808 nm (power of 0.1W, radiant exposure of 1061 J/cm^2^, and energy of 3 J per point, with a 30-second duration at each point, totaling 12 J), adapted from Sierra et al. (2015) [24].

Intraoral irradiation (Table 1) will be performed by placing the laser head directly in contact with four points on the lingual mucosa in the surgical field (alveolus of the tooth to be extracted): At the site where the suture will be performed (middle of the bone socket), on the lingual surface (cervical third), on the lingual surface (middle third), and on the lingual surface (apical third).

**Table 1:** Parameters of intraoral photobiomodulation.

Parameters	Red Laser	Infrared Laser
Wavelength (nm)—Central wavelength	660	808
Operating Mode	Continuous	Continuous
Spectral Bandwidth (nm)	5	5
Average Radiant Power (W)	0,1	0,1
Polarization	Random	Random
Irradiance at the target per point (W/cm^2^)	35	35
Beam Area at the target (cm^2^)	0,002827	0,002827
Exposure Time per Point (s)	30	30
Radiant Exposure at the target tissue per point (J/cm^2^)	1061	1061
Radiant Energy per Point (J)	3	3
Total Radiant Energy (J)	12	12
Irradiated Points	4 points	4 points
Number of Sessions and Frequency	Single session	Single session
Application Technique	contact	contact

J-Joules, W-Watt

Simulated irradiation will be performed at the same points described earlier. This protocol will be applied one hour prior to surgery.

Extraoral photobiomodulation*—*The extraoral photobiomodulation will be applied using a cluster with a contact area of 20 cm^2^ over the insertion point of the masseter muscle.

Extraoral photobiomodulation*—*Extraoral photobiomodulation will be applied using a cluster with a contact area of 20 cm^2^ over the insertion point of the masseter muscle. The cluster will be of the brand Ibramed (Amparo, São Paulo, Brazil), model Antares (small cluster 2). Irradiation will be performed [21, 25] using the cluster of 5 LEDs with a wavelength of 630 nm (power of 0.25 W per LED, radiant exposure of 3 J/cm^2^, and energy of 12 J, with a total time of 48 seconds, totaling 60 J of energy). The same region will then be irradiated with the cluster of 4 LEDs with a wavelength of 850 nm (power of 0.3W per LED, radiant exposure of 2.4 J/cm^2^, and energy of 12 J per point, with a total time of 40 seconds, totaling 48 J of energy). This protocol will be applied one hour prior to surgery (Table 2).

**Table 2:** Parameters of extraoral photobiomodulation.

Parameter	Red LED Vermelho	Infrared LED
Central Wavelength (nm)	630	850
Operating Mode	Continuous	Continuous
Spectral Bandwidth	20nm	35nm
Average Radiant Power (W) per LED	0,25	0,3
Total Power (W)	1,25	1,20
Polarization	random	random
Irradiance at the Target Tissue (W/cm^2^)	0,06	0,06
Beam Area at the Target Tissue (cm^2^)	20	20
Exposure Time (s)	48	40
Radiant Exposure at the Target Tissue (J/cm^2^)	3	2,4
Radiant Energy (J)	12	12
Total Energy per Cluster	60	48
Number of Points Irradiated	5 points	4 points
Number of Sessions and Frequency	Single session	Single session
Application Technique	contact	contact

J-Joules, W-Watt

Individuals who experience any complications during the research period, such as allergic reactions to any of the materials used, an allergic reaction to Paracetamol®, or having taken any other drug not provided, those who, in any way, become unable to attend the appointments on the scheduled dates, relocate to a different city, or simply no longer wish to participate in the research will be excluded from the study but will not suffer any negative consequences in terms of treatment.

The data of all participants will be included in the statistical analysis, described, and discussed, along with any potential major and minor adverse effects.

### Study outcomes

The primary outcome of the study will be:

Edema—three measurements will be taken on the patient’s face with a previously sanitized flexible measuring tape: tragus-pogonion, tragus-labial commissure, and mandibular angle-outer orbital commissure [26]. Measurements will be taken immediately before surgery as well as two and seven days after surgery.

The secondary outcomes of the study will be:

Pain Sensitivity*—*The visual analog scale (VAS) is the most widely used instrument for measuring postoperative pain after oral surgeries with photobiomodulation in the postoperative period [24, 26]. In this study, the VAS will be used immediately after surgery as well as two and seven days after surgery [24, 26] using 10-cm line with "0" (no pain) at one end and "10" (unbearable pain) at the other. Instructions on marking will be provided to the patient by the same operator.

Quantity of pain tablets taken during the period*—*The number of analgesic tablets taken at two and seven days will be counted. All participants will take paracetamol and be instructed to take it every six hours only in the presence of pain [27]. At the beginning of the study, each participant will be provided a paracetamol tablet, which is a drug with purely analgesic effects [28]. At the end of the experiment, the quantity of tablets will be evaluated as a secondary outcome. Patient adherence will be monitored. For such, the patients will be asked to bring the medication blister pack to the appointment to check how it is being used.

Postoperative trismus assessment*—*Spasms in the masticatory muscles (trismus) can limit or even prevent mouth opening after the surgical removal of impacted third molars [21, 23, 29]. This outcome is typically evaluated by measuring the distance between the incisal edges of the upper and lower central incisors using calipers [27, 29]. In this study, previously calibrated evaluators will measure mouth opening in each patient before surgery as well as two and seven days after surgery.

Impact of the surgical procedure on quality of life*—*Two previously calibrated evaluators will ask patients to respond with "yes" or "no" to the following 10 questions two and seven days after surgery, as described by Sierra et al. (2013) [26] and Sierra et al. (2015) [24].

The 10 questions were:

Are you maintaining your social activities normally?

Are you working/studying normally?

Are you maintaining your regular diet?

Do you have difficulty swallowing due to the surgery?

Do you have difficulty tasting food?

Can you chew on the operated side?

Do you have difficulty sleeping due to the surgery?

Did you have difficulty speaking due to the surgery?

Has your appearance changed due to the surgery?

Do you experience nausea due to the surgery?

Dysphagia—The assessment of dysphagia will be conducted two and seven days after surgery through questions [27] and classification on a numerical scale: (0) total absence of dysphagia; (1) dysphagia for solid foods; (2) dysphagia for any liquid or solid food.

Assessment of the presence and intensity of hematoma/ecchymosis*—*The presence of hematoma/ecchymosis will be assessed by measuring the largest diameter of color changes on the skin of the cheek and submandibular region at two and seven days after surgery. The measurement will be performed by a previously calibrated evaluator, who will classify the occurrence of this event in four categories: (1) non-existent; (2) largest diameter less than 4 cm; (3) largest diameter between 4 and 10 cm; and (4) largest diameter greater than 10 cm, as described by Bjornsson et al. (2003) [30].

### Statistical analyses

Initial descriptive analyses will be performed for all variables measured in the study, both quantitative (mean and standard deviation) and qualitative (frequencies and percentages). Normality tests will then be conducted to determine the appropriate statistical tests for specific analysis. Subgroup analysis will be performed: treatment x sex interaction [31]. A significance level of 5% probability or corresponding p-value will be adopted for all tests. All analyses will be conducted using the statistical software SPSS for Windows, version 9.1. Interim analysis was not planned.

## Discussion

For the management of pain and swelling following third molar extractions, some authors recommend the pre-emptive use of anti-inflammatories. However, conclusive evidence regarding the efficacy of pre-emptive use remains elusive. In a 2018 systematic review addressing optimal dosages and administration routes for corticosteroids in lower third molar extractions, indicated an absence of well-established standards for the ideal route, type, and dosage. Nonetheless, preoperative corticosteroid administration through submucosal injection demonstrated effectiveness in mitigating edema, pain, and trismus post-surgery (Larsen et al., 2018) [10]. In 2019, a meta-analysis found that methylprednisolone, irrespective of administration route, significantly alleviates immediate postoperative edema, albeit with no discernible impact in the subsequent days following lower third molar extractions. Oral administration or intra-masseter application appeared promising in reducing pain and trismus during the immediate postoperative period. Notably, oral administration demonstrated potential in mitigating late-phase pain, while intra-masseter administration effectively controlled trismus. Despite these findings, researchers underscored the imperative for additional controlled and randomized clinical trials to fortify evidence pertaining to methylprednisolone prescription in lower third molar extractions [12]. A 2019 systematic review with meta-analysis concluded that corticosteroids exhibit greater efficacy than placebos in managing pain and trismus associated with the postoperative period of lower third molar extractions, particularly when employed preventively or pre-emptively. The route of administration minimally influenced outcomes, except for the submucosal route. Continuation of corticosteroid use post-surgery did not yield additional positive effects in postoperative comfort [13]. Nevertheless, a 2021 systematic review with meta-analysis by Singh et al. [14] indicated that the use of systemic corticosteroids, even in oral surgeries involving trauma, rests on weak scientific evidence, necessitating further studies to substantiate its recommendation.

Photobiomodulation (PBM) has emerged as a viable alternative for postoperative management in third molar surgeries, effectively controlling pain, swelling, and trismus [17, 18]. Two studies have highlighted the preventive efficacy of PBM, specifically with infrared wavelength, in lower third molar extractions [19, 20]. However, the combined preoperative application of PBM has not been explored to date. Considering the positive outcomes observed with PBM, it is plausible that pre-emptive application using both red and infrared LED, both extraorally and intraorally, could prove effective in reducing postoperative swelling in lower third molar extractions. Consequently, PBM stands as a non-pharmacological alternative to enhance postoperative comfort following impacted lower third molar extractions.
